# Systems for Muscle Cell Differentiation: From Bioengineering to Future Food

**DOI:** 10.3390/mi13010071

**Published:** 2021-12-31

**Authors:** Kah-Yin Lee, Hui-Xin Loh, Andrew C. A. Wan

**Affiliations:** Singapore Institute of Food and Biotechnology Innovation, 31 Biopolis Way, #01-02, Nanos, Singapore 138669, Singapore; Lee_Kah_Yin@sifbi.a-star.edu.sg (K.-Y.L.); Loh_Hui_Xin@sifbi.a-star.edu.sg (H.-X.L.)

**Keywords:** muscle cell differentiation, tissue engineering, microsystems, biomaterials

## Abstract

In light of pressing issues, such as sustainability and climate change, future protein sources will increasingly turn from livestock to cell-based production and manufacturing activities. In the case of cell-based or cultured meat a relevant aspect would be the differentiation of muscle cells into mature muscle tissue, as well as how the microsystems that have been developed to date can be developed for larger-scale cultures. To delve into this aspect we review previous research that has been carried out on skeletal muscle tissue engineering and how various biological and physicochemical factors, mechanical and electrical stimuli, affect muscle cell differentiation on an experimental scale. Material aspects such as the different biomaterials used and 3D vs. 2D configurations in the context of muscle cell differentiation will also be discussed. Finally, the ability to translate these systems to more scalable bioreactor configurations and eventually bring them to a commercial scale will be touched upon.

## 1. Introduction

With an impending food crisis and irreversible climate change looming at close quarters, conventional animal agriculture and its produce of livestock meat must be supplemented, if not replaced, by more sustainable, alternative sources of protein. As animal meat is rich in protein and serves as an important source of nutrition for people around the world it is unreasonable to expect the consumption of meat to stop instantly. Nevertheless, the development of alternative protein sources that closely resemble meat—cell-based, cultured, or cultivated meat in particular—would go a long way towards reducing the harmful impacts of conventional animal agriculture and serve as a pragmatic approach towards sustainability.

While cultivated meat is seen as a promising development for future food many questions remain unanswered. In animals cells proliferate and then differentiate into mature tissues under controlled microenvironmental conditions. To obtain cultivated meat in vitro cells are first propagated from suitable source cells to obtain the numbers and volume necessary to constitute a meat product. One would believe that the differentiation of myoblasts into myotubes and myofibers is essential to obtain the desired texture of the final product; would this same requirement for differentiation extend to nutritional quality? Are scaffolds, such as those used in tissue engineering, required to promote muscle differentiation in the production of cultured meat? Mosa Meat, Upside Foods, and Eat Just are a few companies that have successfully grown cell-based meat in their labs. At present their cultivated meat is in a comminuted form, as seen in products such as nuggets, hot dogs, and burger patties, where scaffolds can be dispensed with. On the other hand, the long-term goal is to develop three-dimensional (3D) complex structured meats products such as steaks, fillets, and bacons, which may necessitate scaffolds to define the structure of the meat product. How would the initial mechanical properties of the scaffold affect differentiation and thus the development of texture, eventually influencing the quality of the final product?

In this article we will review the literature and discuss systems for muscle cell differentiation in the context of practicing the same methods towards the objective of making cultured meat products. We will first review the structure of muscle, the types of source cells that can be differentiated, and the different routes for differentiation. Next, we discuss the various biological, physicochemical, and mechanical factors that affect muscle cell differentiation. One important factor to consider when developing 3D structured meats is the scaffold, which is used to house the cells outside of animals. Associated with the scaffold is the material’s aspects—the chemical nature of the biomaterial from which it is constructed, its mechanical properties, and its porosity, in addition to how these factors affect the differentiation of cells growing in or on the scaffold. In this review we will also discuss the advantages of hydrogels in providing a suitable 3D environment to support differentiation and the structuring of whole muscle tissue. Next, we discuss the use of mechanical and electrical stimuli in influencing muscle cell growth and differentiation. Finally, for cultured meat to be a feasible alternative to the conventional production of meat, systems for muscle differentiation at the bench scale would have to be scaled-up in order to achieve commercial viability. We will discuss here how the knowledge and techniques that have been developed at the microscale can be employed to the best effect.

## 2. Recapitulating Muscle In Vitro

### 2.1. Muscle Structure and Cultured Meat

There are three different muscle types classified under muscle tissue—smooth, cardiac, and skeletal muscle, with the latter being the most commonly used for cultured meat. The meat that we consume comprises proteins and tissues of the musculoskeletal system; to obtain cultured meat muscle cells are essentially cultured in vitro to recapitulate native muscle. Thus, an understanding of skeletal muscle biology would help us to recognize the associated challenges. On a macroscopic scale skeletal muscle is made up of roughly 90% muscle fibers, with the remaining 10% comprising adipose and connective tissues. In spite of the fact that adipose and connective tissues make up the smaller percentage of meat they are still important to consider as the tenderness, juiciness, and flavor of meat is highly dependent on these components [1]. Hence, the combination of muscle fiber and adipose tissue determines the appearance and taste of the end product. Another key consideration in replicating conventional meat is simulating its texture and structure. In contrast to texture, the structural property of meat is independent of its composition [2] and can be modulated accordingly by its surrounding environment, i.e., the scaffolding in which the cells are housed for growth. There are also bones, blood vessels, and nerves surrounding the skeletal muscle tissues. However, it is challenging to mimic every component in the musculoskeletal system and in fact unnecessary, as cultured meat is often compared to the dehydrated and exsanguinated meat products sold in the supermarkets [3]. The focus should thus be on the culturing of skeletal muscle and adipose tissue as the minimal required components in developing cultured meat for consumption.

**Figure 1:**
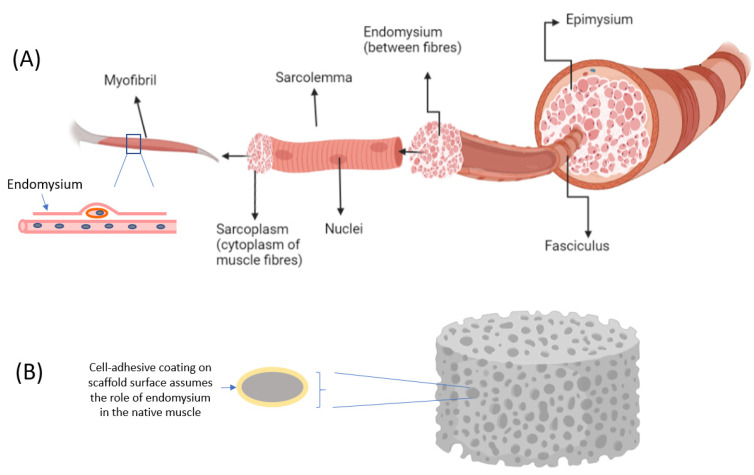
The role of the scaffold (**B**) in guiding muscle cell differentiation and muscle fiber development is analogous to that of muscle connective tissue layers (**A**). The extracellular matrix of the endomysium plays an important signaling role in the regeneration of muscle fibers (adhesion, differentiation, proliferation, and inhibitory cues). In scaffolds the role of regulating muscle maturation is assumed by the coatings or surface modifications of the scaffold. Collectively, the endomysium, perimysium, and epimysium define the different levels of muscle organization and structure, a role which is also assumed by tissue scaffolds.

Skeletal muscle is a complex tissue structure made up of multinucleated cells that are elongated and tubular in shape, known as myocytes, myofibers, or muscle fiber. Each muscle fiber measures approximately 10 to 100 μm in diameter and ranges from a few millimeters to several centimeters in length [2]. The bundled muscle fibers form one fascicle and many fascicles come together to form skeletal muscle. Adipose tissues are a collection of loose adipocytes deposited within the muscle bundles [4]. Meanwhile, every skeletal muscle is enclosed by three layers of connective tissues, namely the epimysium, perimysium, and endomysium. These tissues contribute significantly to the formation of muscle structure and are needed for muscle differentiation and development. When designing systems for muscle cell differentiation it may be useful to draw an analogy between the role of these connective tissues and the role of the scaffold (Figure 1). For example, the endomysium that surrounds and comes into contact with each muscle fiber is a source of the extracellular matrix (ECM), which provides the cues that guide muscle development. Collectively, the connective layers also provide mechanical support for muscle cells at different levels, at the same time separating muscle fibers within the muscle. Scaffolds can thus be designed to recapitulate the structure of muscle in vitro.

### 2.2. Cell Types Used

Prior to undertaking the culturing of cells and tissues for cultured meat, cell sourcing is a key technical component to be evaluated. Whether one works with cell lines or primary cells, both require further bioprocessing optimization in order to reach an industrial scale. The chosen cell source and bioprocess depend on the desired conditions and parameters to be achieved, such as the suspension growth, growth rates, and differentiation capacity. In all cases a prerequisite for any cell source is the capacity to proliferate and differentiate into skeletal muscle when grown and cultured in an in vitro environment, especially one where animal serum and other animal-derived components have been eliminated.

Stem cells are desirable candidates to be employed for cultured meat owing to their ability to self-renew and propagate indefinitely, as well as their ability to differentiate into many cell types. Stem cells from different stages of development are studied for the production of cultured meat [5] and can be broadly classified into embryonic stem cells (ESCs) and adult stem cells. ESCs are acquired from the inner mass of blastocysts found in an early stage embryo. They are pluripotent in nature [6], i.e., able to differentiate into the three primal germ layers, namely the ectoderm, mesoderm, and endoderm. The method of cellular reprogramming involves the introduction of transcription factors into somatic cells, such as dermal fibroblasts, resulting in induced pluripotent stem cells (iPSCs) with pluripotent characteristics that are very similar to ESCs. [7,8]. Upon terminal differentiation into other tissues, pluripotent cells (both ESCs and iPSCs) eventually lose their pluripotency [7,9,10]. In terms of differentiation into specific musculoskeletal cell types relevant to cultured meat, pluripotent cells are challenging to work with as they are extremely sensitive to their growth environment. A further disadvantage of pluripotent cells in cultivated meat applications is the risk of residual undifferentiated cells in culture, which could give rise to teratomas [11].

In contrast to iPSCs, adult stem cells do not exhibit pluripotency but display multipotency instead. Many studies have demonstrated the structural and regeneration capabilities of adult cells, whereby they self-renew to replenish dying cells and aid in the regeneration of damaged cells [12]. Adult stem cells are also known as resident stem cells, which are populations of undifferentiated stem cells found in various tissues throughout the body. The main resident stem cell of skeletal muscle tissue is the muscle stem cell, also known as the satellite cell. These cells are located between the sarcolemma and basal membrane of each muscle fiber (myofiber) (Figure 1). The skeletal satellite cells remain in a quiescent state until there is a trigger that activates them to a specific location in response to muscle injury or diseases [13]. The activation will return satellite cells to the start of the cell cycle to proliferate and differentiate into embryonic progenitor muscle cells called myoblasts, which can in turn be differentiated into mature muscle tissue. Myoblasts then undergo myogenesis followed by muscle-specific gene expression for myoblast fusion, before terminal differentiation into matured multinucleated myotubes and, finally, myofibers [14]. This sequence of differentiation was used to develop the prototype of the first cultured hamburger [15].

As adult stem cells, in particular myosatellite cells, will only mature into their own specific lineages upon differentiation, the targeted cell type is more easily achieved and undesired cell types are avoided. However, myosatellite cells require a scaffold substrate for anchorage, as opposed to pluripotent cells, which can be propagated in a suspension culture [16]. As with pluripotent cells they can be rather expensive to grow and maintain, requiring specific growth factors to direct them into the specific differentiation pathways. Hence, a considerable amount of effort is required to optimize and adapt the processing of these cells to a manufacturing scale [17]. In contrast to skeletal muscle tissue, the culturing of adipose tissue does not require any adherent substrate. In vivo adipose tissue is in the form of lipid droplets, and due to the nature of their buoyancy mature adipocytes do not require attachment to any surface but can be cultured while suspended in the cell medium [18].

Another reprogramming technology called lineage reprogramming, also known as transdifferentiation, is a process where specialized cell types can be directly transformed from one lineage to another lineage without going through the pluripotent state. Naoki Ito and co-workers [19] have identified the reprogramming transcription factors to generate skeletal muscle progenitor cells directly from fibroblasts, while Wei Wu and colleagues have demonstrated that fibroblasts can be reprogrammed into adipogenic stem cells [20].

Both skeletal muscle stem cells and adipogenic stem cells can be acquired easily by taking a cell sample directly from live animals [21]. Certain religious practices specify methods for the slaughtering of animals: halal processing for Islam and kosher processing for Judaism, for example, where the animals are not handled until they are dead [22]. In compliance with these practices it is also possible to acquire cells from animals that have been recently slaughtered, where the tissues and their content of cells are still viable. Besides ‘muscle’ meats, organ meats such as intestines and foie gras (goose liver) can be recreated by obtaining cells from specific organs of animals. At the same time researchers and scientists are also exploring insects, mollusks, and seafood as potential sources for cultivation.

Meanwhile, extensive studies can be done in the lab to understand the features of animal tissues that are important when recreating meat. The murine C2C12 and rat L6, both derived from thigh muscle, are immortalized myoblast cell lines that are commonly used as cell models to recapitulate the physiology of skeletal muscle tissue in vitro [23]. This allows the important features of skeletal muscle to be studied [24]: for example, how topological cues can align myoblasts in addition to the effects of biomaterials and biochemical as well as physical factors on their differentiation. C2C12 cells are often used to investigate the effects of contraction stimuli while L6 cells are used for studying metabolism and nutritional signals [25,26,27]. Both C2C12 and L6 myoblast cells can proliferate under high-serum conditions and differentiate into myotubes in low-serum conditions. C2C12 cells can be differentiated into myotubes and, henceforth, myofibers, recapitulating the development of muscle tissue in vivo, making them a useful cell line for preliminary studies and investigations.

## 3. Cell Adhesion, Alignment, and Influence of the Physical Environment

### 3.1. Cell Adhesion

**Figure 2:**
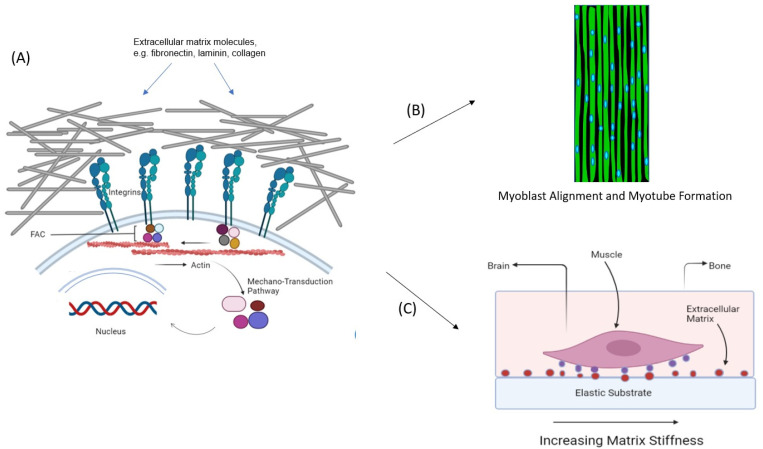
(**A**) Muscle cell adhesion is mediated by integrin receptors on the cell membrane, which bind to ligands present in the extracellular matrix. This results in signaling via the mechanotransduction pathway, which leads to changes in gene expression and the modification of the cell phenotype. Cell adhesion is important for two reasons: (**B**) it enables myoblasts to follow geometrical cues, leading to their alignment as well as myotube and myofiber formation; (**C**) it allows stem cells to sense the mechanical properties of the underlying substrate and differentiate accordingly into specific tissue lineages. Mesenchymal stem cells have been shown to differentiate into the muscle lineage when cultured on polyacrylamide substrates with elastic moduli ranging between 8–17 kPa.

Cell Adhesion Is Mandatory for Cell Survival, Especially for Adherent Cell Types [28,29]. Adhesion is critical for muscle maturation as it mediates the effects of topographical cues and mechanical factors on cell differentiation (Figure 2). To mediate cell adhesion in native tissue, integrin receptors on the surface of cells bind to corresponding ligands present in extracellular matrix molecules such as collagen and noncollagenous glycoproteins, fibronectin, and laminin [30,31]. Thus, the adhesion function relies on two components— receptors on the cells, which are mainly from the integrin family, and ligands on the extracellular matrix (ECM) proteins that surround the cell. There is a close interplay, involving molecular interactions, between the cells and their ECM. Take for example the case of fibronectin, which is secreted by the cells in a compressed and unfolded form. The fibronectins bind with the cell surface α5β1 integrins, transforming them into functionable dimers. A high concentration of these activated dimers will simulate interconnections between them as well as with other molecules from neighboring cells, forming a network throughout the matrix. The matrix assembly domain of fibronectins induces the gathering of other proteins, such as collagens, within the ECM for the formation of fibers [32,33]. Similarly, laminins aid in the cell adhesion process by interacting with the integrin receptor, forming networks of web-like structures that can resist tensile forces [34] (Figure 2).

For in vitro culturing, several strategies exist to imbue surfaces or scaffolds with moieties that mediate cell adhesion and promote cell spreading. Quite often, cell recognition motifs that act as ligands to cellular receptors are employed. For example, Ary-Gly-Asp (RGD), Tyr–Ile–Gly–Ser–Arg (YIGSR), and Ile-Lys-Val-Ala-Val (IKVAV) are cell recognition motifs derived from corresponding sequences of amino acids in ECM proteins such as fibronectin, collagen, and laminin. RGD peptides can be incorporated directly or be chemically crosslinked with the biomaterial scaffold [35,36]. However, up to the present time, RGD peptides have yet to be approved for consumption. In place of RGD and other cell recognition motifs, the full animal-derived proteins such as collagen and gelatin (hydrolyzed collagen) themselves can be used in systems to support muscle cell differentiation [37]. These can be obtained in food-grade states; however, their animal source is not aligned with the idea and rationale of developing cultured meat. Alternatively, plants and fungi have also been reported to express proteins with the RGD sequence, which may potentially be used as non-animal-derived substitutes [38,39]. With or without the use of ECM proteins and cell recognition motifs, methods such as plasma treatment or ion beam deposition can be employed to functionalize scaffolds and alter their surface properties in order to promote cell activity [40]. For example, the treatment of electrospun poly(L-lactide) microfibrous scaffolds with mild Ar or Ar-NH3/H2 plasmas enhanced the attachment, growth, and infiltration of bovine aorta endothelial cells and smooth muscle cells [41]. Nano-coated glass and titanium surfaces produced by ion-beam-assisted deposition enhanced bone-associated gene expression and cell differentiation of osteoblasts [42].

### 3.2. Muscle Cell Alignment

The architecture of skeletal muscle is highly governed by the alignment of matured muscle fibers. Under a microscope skeletal muscles appear striated with nucleated fibers in repeating dark and light bands due to the myosin and actin myofilaments, respectively. These myofilaments are long cylindrical structures found along the length of myofibrils within each muscle fiber. Assembled in a repeating arrangement to form sarcomeres, they are responsible for highly coordinated activities such as muscle contraction. For the accurate transmission of the forces applied myofibers must be aligned accordingly; the skeletal muscle fibers are usually aligned with their native extracellular matrix (ECM). In fact, unaligned muscle fibers will give rise to muscle dysfunctions such as myotonic dystrophy, which is a common muscle disease whereby patients experience progressive skeletal muscle degeneration, leading to severe disability [43]. In engineering muscle tissue muscle cells should also be pre-aligned by the scaffold matrix to promote muscle maturation. In fact, it has been shown that myotube fusion and myofiber maturation are promoted on engineered aligned microstructures in the form of meshes, lines, and fibers [44].

Cells such as murine myoblasts, endothelial cells, and fibroblasts can be provided with geometrical cues, whereby they align themselves in an elongated manner, the effectiveness of such cues depending on how the biomaterial scaffold is fabricated and the type of biomaterial itself [45]. Different biofabrication techniques such as micropatterning, micromolding, electrospinning, and 3D bioprinting can provide these topographical guidance cues for myoblast alignment. Fernandez et al. have successfully fabricated 3D skeletal muscle microtissues by encapsulating transdifferentiated myoblasts derived from myotonic dystrophy type 1 (DM1) patients in hydrogels. These hydrogels were obtained by photomold patterning of light-sensitive gelatin methacryloyl-carboxymethyl celloluse methacrylate (GelMA-CMCMA) monomers, and exhibited a microstructured topography that guided myoblast differentiation into highly aligned multinucleated myotubes [45]. Electrospinning may be advantageous for skeletal muscle tissue engineering on account of its ability to provide aligned fibers that direct myotube formation. Soliman et al. demonstrated the elongation of C2C12 cells myoblasts cultured on aligned, electrospun fiber networks [46], while electrospun polycaprolactone fibers worked in synergy with 5-azacytidine, a reprogramming factor, to promote human mesenchymal stem cell differentiation towards the muscle lineage [47]. Three-dimensional bioprinting is a relatively new tool for skeletal muscle tissue engineering. Costantini and co-workers achieved the formation of highly aligned multinucleated myotube fibers by using 3D bioprinted hydrogel fibers constructs, which were unidirectionally aligned with C2C12 cells after 21 days of in vitro culturing [48].

### 3.3. Physical Environment

The binding of integrin receptors and cell recognition motifs will induce the downstream mechanotransduction signaling pathway and, consequently, control how the cell behaves and functions (Figure 2) [49,50]. In essence, integrins function by transmitting information from the ECM to the actomyosin cytoskeleton in the cell through the focal adhesion complexes [51]. The amplitude of the forces generated depends on the mechanical properties of the ECM, one important parameter of which is matrix stiffness. Skeletal muscle tissue has an intermediate range of matrix stiffness, with a Young’s modulus value of approximately 10 kPa, while hard bone tissue has a higher Young’s modulus value of approximately 30 to 45 kPa [52]. If the Young’s modulus is not within the range of the specific tissue, the cells will not be able to take on cues from their ECM. In the same vein, matrices should be engineered with the appropriate Young’s modulus value, thus controlling the fate of stem cells when recreating specific tissue. In a landmark study, Engler et al. showed that mesenchymal stem cells differentiate into specific lineages in accordance with the stiffness of the substrate upon which the cells are cultured [53]. Mesenchymal stem cells cultured on polyacrylamide substrates with Young’s moduli ranging between 8–17 kPa were shown to differentiate into a skeletal muscle phenotype, which agrees well with the stiffness of the native muscle microenvironment (Figure 2). Such an effect is also observed for cells cultured in 3D matrices [54]. Interestingly, by sensing the stiffness of the surrounding matrix stem cells can contextually reorganize the ECM to create a local niche. This mechanosensing mechanism involving forces from the cytoskeleton to the adhesive bond is influenced by the ECM composition as well as by the expression of particular subsets of integrin heterodimers [55].

## 4. Scaffolds to Guide Muscle Tissue Development

For cells to grow in an in vitro setting it is essential to engineer a natural scaffold framework that resembles the native microenvironment for the cells to adhere onto. The scaffold will follow through and support the whole cellular process from the stage of cell seeding until the end product. The viability of cells in a scaffold is crucial and depends on the biocompatibility of the scaffold as well as its physical properties. In addition to being edible, an ideal scaffold for cultivating muscle cells should allow for the efficient exchange of gases, nutrients, and waste. The native microenvironment can be recreated successfully if the stiffness and eventual protein composition of the native ECM can be mimicked. This promotes more cell–cell and cell–matrix interactions, which enable cells to proliferate and differentiate desirably to replicate the specific tissue type. Scaffolds can also enhance the viability and maturation of skeletal muscle cells by providing topographical cues [45].

Cell adhesion and differentiation also depend on the pore size of the scaffold, which should be in the appropriate range. Pore sizes that are too small result in limited cell migration and the formation of a cellular capsule around the scaffold edges, whereby the diffusional exchange of gases and nutrients as well as the removal of waste will be restricted. On the other hand, if the pore size is too big the surface area will be smaller, limiting cell adhesion [56]. In one study, the effect of the pore size of collagen–glycosaminoglycan (CG) scaffolds on cell adhesion and viability was investigated. It was shown that as the mean pore size increased the number of viable cells attached to the CG scaffolds decreased [57]. Additionally, different cell types require different optimal pore sizes to support cell activities. Bone marrow mesenchymal stem cells, a potential myogenic precursor, showed optimal adhesion and proliferation in scaffolds of a pore size of ~200 µm [58]. An important design parameter for fibrous scaffolds is the fiber size. Larger-diameter polyester fiber matrices supported significantly higher proliferation and alignment of C2C12 myoblasts, indicating a curvature sensing ability of the cells [59].

Scaffolds should ideally mimic the natural in vivo environment of skeletal muscle cells. Scaffold fabrication techniques such as electrospinning, mold cast systems, injectable systems, and 3D extrusion printing have been used in muscle tissue engineering, but there are limitations associated with each technique. Where the electrospinning technique is of concern, the small pore size, thinner-than-expected cell thickness, and low z resolutions make it difficult for cells to infiltrate and subsequently proliferate and differentiate. Similarly, the mold cast system and injectable systems pose problems such as limited control over the pattern porosity and properties of the scaffold. Finally, for 3D extrusion printing, issues such as the low viscosity of bioinks, shear stress faced by cells from fluids, as well as encapsulated fibers limiting nutrients to the cell are some examples of why the method still needs improvement [44]. Three-dimensional printing, nevertheless, is an enticing option due to its control of the precision in the deposition and assembly of the selected materials and cells, in turn improving cell–cell and cell–extracellular matrix interactions [60]. It was demonstrated in one study that the meticulous selection of the nozzle for the 3D printer allowed the cells to be treated with shear stress and guided them in the direction of the printing, thereby promoting myotube formation [61].

## 5. Systems for Muscle Cell Differentiation

### 5.1. D vs. 3D Systems

Conventionally, cell culturing is achieved in laboratories with a simple two-dimensional (2D) system where tissue culture flasks and Petri dishes are used as the adherent mechanical support for cells. Two-dimensional cell culturing is commonly used to study cell morphology and the influence of topographical cues on cells. Even with this being the case, when cells are transferred to a 2D environment from their native tissue they tend to lose their native phenotype and morphology, causing a change in their behaviors, functions, and gene expression. Due to the lack of structural cues 2D cultured cells are anchored firmly onto the bottom of substrates, forming a monolayer of cells with restricted access to the cell culture medium, which contains all the essential ingredients needed for cell growth [62,63]. Over a period of time 2D cell culturing becomes unsustainable. It is certainly the case that 2D cell culture systems do not emulate the in vivo environment of the native complex tissue of skeletal muscle accurately, whereby cells of distinctive lineages require various biochemical and physical factors in different 3D compartments for interactions [64]. Cells in 2D systems hardly communicate with each other and there are barely any cell–matrix interactions, resulting in slower proliferation, limited differentiation, and the failure to form cohesive tissue features, such as tubular and cyst structures, found in epithelial tissue.

To better mimic the behavior of three-dimensional (3D) native ECM in vitro cells should be surrounded by a 3D scaffold matrix system, which can be constructed with suitable biomaterials, to better represent important tissue features [65,66]. The hydrogel form is often used in tissue engineering applications to provide a 3D environment. Hydrogels are made of crosslinked polymers that have a specific affinity for water owing to the presence of a hydrophilic functional group in their polymeric backbone, such as amide (-CONH-, -CONH_2_), amine (NH_2_), hydroxyl (-OH), and sulphate (-SO_3_H). When crosslinked the 3D networks prevent the hydrogel from dissolution while having the swelling capability to absorb water in large quantities, several times their own weight [67]. This high-water-content attribute is akin to various native tissues, which is favorable when designing a biomaterial scaffold [68,69]. Other hydrogel characteristics, such as their microporous structure, soft mechanical properties, and biocompatibility [70,71,72], have seen them be employed in applications such as the delivery of bioactive molecules, agents for filling vacant spaces, and, importantly, as 3D cell support structures for tissue formation [73]. Hydrogels are considered as smart materials, whereby they are responsive to their external environment; any changes in temperature, light, chemical, pH, or ionic strength cause them to degrade or swell, eventually changing their structural or mechanical properties [74,75]. In addition, the physical appearance of hydrogels, such as their structure, shape, and size, can be tuned accordingly to be used in different conditions. Due to their easily modifiable properties and biocompatibility, hydrogel materials can be implemented to design different types of scaffold structures as supporting systems for tissue formation, even for the complex tissue of skeletal muscle. In the latter application the main requirement is the ability to adjust the hydrogel’s mechanical properties to conditions that recapitulate skeletal muscle’s niche, as discussed in Section 3.3.

For skeletal muscle tissue engineering, hydrogel scaffolds can be designed into 3D forms of fiber, spheres, or even bulk constructs. Since each skeletal muscle cell is a muscle fiber designing scaffolds in fibrous forms will enhance myogenesis, the high aspect ratio of fibrous hydrogels supporting muscle maturation by providing the necessary geometrical cues for the alignment of muscle cells. Mirani et al., 2020 introduced the use of fibrous hydrogels with solid grooves and hollow features that were fabricated using a textile-technology-like embroidery procedure. When C2C12 myoblasts were cultured on the grooved fibers the cells were aligned in the direction of the grooves, promoting myogenic differentiation in a material-dependent manner [76]. In contrast, cells cultured on smoother fibers resulted in an aimless orientation. When cell-laden hydrogels are deposited in a layer-by-layer manner even tissues with a complex geometry can be fabricated. Mozetic et al. presented a bioprinting methodology with the application of additive manufacturing (AM) to tissue engineering, using an alginate/pluronic hydrogel [77]. C2C12-myoblast-containing constructs were printed where cells were aligned within fibers in the direction of deposition, leading to an enhancement in myogenic gene expression.

### 5.2. Natural vs. Synthetic Hydrogels

Depending on their formulation hydrogel polymers can be categorized into either synthetic or natural materials. Some common polymeric synthetic materials include poly (propylene fumarate) (PPF), poly (ethylene oxide) (PEO), poly(vinyl alcohol) (PVA), and poly(propylene fumarate-co-ethylene glycol) (P(PF-co-EG)) [78]. Synthetic hydrogels may have superior modifiability in terms of mechanical properties [79]; however, they usually lead to inferior cellular functions such as cell adhesion, proliferation, and differentiation, leading to the suboptimal formation of tissues, eventually requiring bioactive cues to improve cellular function [68]. Often, for skeletal muscle tissue engineering, several synthetic hydrogels would be used together. Xu et al. used a mixture of thermosensitive injectable hydrogels (acrylic acid (AAc), N-isopropylacrylamide (NIPAAm), and degradable macromer 2-hydroxyethyl methacrylate-oligomer) to encapsulate rat bone marrow mesenchymal stem cells (MSCs) in hydrogels with elastic moduli of 20 and 40 kPa, where myogenic differentiation and muscle regeneration were enhanced in the stiffer material [80].

Natural hydrogels have their own advantages, such as higher biocompatibility and biodegradation by enzymes, leading to biocompatible byproducts [81]. When it comes to choosing natural hydrogels for cultured meat application animal-derived biomaterials such as collagen are generally avoided. The preferred hydrogels are those sourced from biopolymers/polysaccharides that are found abundantly in nature, such as agarose, alginate, chitin, and chitosan [82,83,84,85,86].

Natural hydrogels have been tested for their ability to support in vitro myogenesis, focusing on their tensile properties and degradation stability [87]. In other studies a mixed hydrogel of synthetic and natural polymers has been employed. Fuoco et al. demonstrated the formation of myotubes by adult skeletal-muscle-derived pericytes after 30 days of culturing in PEG–fibrinogen hydrogels [88], while Rich et al. used a mixture of alginate methacrylate and poly (ethylene glycol) diacrylate (PEGDA) hydrogels to fabricate skeletal muscle tissue [89].

Both synthetic and natural hydrogels can be formed by polymerization mechanisms, through the chemical or physical crosslinking of water-soluble monomers or macromers into gel-like structures. Chemical crosslinking involves polymerization through covalent bonding, while physical crosslinking involves physical interactions through secondary bonding, such as hydrogen bonding, ionic reactions, and also through crystallization. The formation of hydrogels can also be initiated by the incorporation of biological agents such as enzymes or amino acids. Both chemical and physical crosslinking systems can be designed to take place at a neutral pH and physiological ionic concentrations, allowing cells to be encapsulated without exposing them to toxic agents [90]. Various ECM molecules such as growth factors and cell-adhesive molecules can be incorporated into hydrogels for their functionalization [91,92].

## 6. External Stimuli for Muscle Cell Differentiation

In addition to the adhesion and alignment of cells that can be achieved by the engineering strategies discussed in the previous sections, further benefits for muscle cell differentiation could be accrued by applying the mechanical stimuli that they would experience in vivo. The application of these forces, especially contractile force, simulates the conditions required for cell proliferation, differentiation, and further muscle maturation [93]. Hydrostatic pressure, mechanical strain, and fluid shear stress are some examples of mechanical stimuli that have been well-documented over the decades [94].

A common form of mechanical stimulus that can be applied towards cell differentiation is cyclic strain. In the human body cyclic strain promotes the regeneration of musculoskeletal cells due to continuous movement of the limbs. Experiments have demonstrated that a frequency below 1 Hz would induce stretching of cells, leading to their efficient proliferation and differentiation. In terms of strain a wide variety of stimulus regimes have been used, with a consensus that uniaxial strain is more beneficial than biaxial strain for myogenesis at strain amplitudes of 10–15% [95]. Additionally, mechanical loading should be applied on cells in a 3D environment as studies have shown that this mimics mechanical loading in natural systems [96]. Under appropriate conditions the 3D loading of cells allows them to recognize mechanical stimulation as cell–ECM interactions, which results in the precise differentiation desired in the reconstruction of native muscle [97]. Ramey-Ward et al. applied cyclic strain by means of gold nanorods coated with a light-responsive hydrogel coating, on which myoblasts were attached. The stimulation of integrin receptors that mediate cell–ECM interactions was shown to enhance myogenesis [98]. Considering the potential application of scaffolds for skeletal muscle regeneration, the mechanics of fibrous, porous cylindrical scaffolds under the action of external cyclic strains have been modeled [99]. Cyclic strain can also be effectively applied on cells cultured in hydrogel scaffolds. For example, Chiang et al. demonstrated how light-driven cyclic bending motions of hydrogels composed of polypeptides and graphene oxide materials could be used to induce and enhance myogenic gene expression [100]. The combined effects of both cyclic strain and ultrasound on muscle cell regeneration have been studied by Kang and his coworkers for tendon tissue engineering. Three-dimensional scaffolds of polycaprolactone (PCL) and poly-L-lactic acid (PLLA) were constructed using the salt leaching method, seeded with MC3T3-E1 pre-osteoblasts, and subjected to the combined stimuli, which promoted efficient cell maturation [101]. In an indirect application of mechanical stimuli Hicks et al. applied cyclic short-duration stretches followed by acyclic duration stretches on fibroblasts, which produced IL-6 that in turn induced myoblast differentiation in fibroblast–myoblast co-cultures [102].

Apart from mechanical stimulation, electrical stimulation can also be used for cell proliferation and differentiation. In the absence of a scaffold structure the combined effects of electrical and mechanical stimulation were shown to promote the initial stages of cell proliferation [90]. Using a commercial cell culture stimulation device, Je et al. investigated the effects of electrical stimulation on rat L6 myoblasts. Upon monitoring the expression of myogenic markers using qPCR and immunochemistry it was deduced that electrical stimulation results in myogenic differentiation by suppressing the expression of small GTPases [103].

In another study Sirivisoot and Harrison demonstrated skeletal myotube formation by using electrospun polyurethane and carbon nanotubes both with and without the influence of electrical conductivity [104]. Fluid shear stress has been shown to induce the differentiation of cardiomyocytes and smooth muscle cells via mechanotransduction [105,106], while the same has yet to be demonstrated for skeletal muscle. On the other hand, the effect of cyclic strain on cardiomyocyte differentiation has been widely established and employed for the engineering of cardiac tissue [107,108].

## 7. Scaling-Up Muscle Differentiation for Cultivated Meat

Translating the cultured meat effort from bench-scale experiments to large-scale commercial application begs the question of how the discussed microscale concepts, such as adhesion, topographical cues, matrix stiffness, and the various external stimuli, can be transferred to the macroscale in order to realize the efficient differentiation and maturation of muscle cells. In recent decades much insight in muscle tissue engineering has been gained from the field of bioengineering; however, as the volume of production and sales that is anticipated for cultivated meat is much larger than that for biomedical implants the upscaling of muscle cell proliferation and differentiation will be key. Terming the broad array of materials that can be used to support cells in large-scale culturing as cell culture supports, these can be further classified into microcarriers for a suspension culture in 3D stirred tank reactors and scaffolds that can accommodate a range of other bioreactor configurations.

### 7.1. Microcarriers

Following the pharmaceutical industry, a likely configuration for scaled-up cultivated meat production would be suspension cultures in bioreactors. Some of the cell types relevant to cultivated meat, such as muscle cells, are anchorage-dependent, thus necessitating the use of microcarriers (MCs) in suspension bioreactors. Three different scenarios have been postulated for large-scale meat production using MCs—the use of MCs as substrates that adhere to cells temporarily and get separated from cells through the removal process, MCs which dissolve once the cells successfully differentiate and proliferate, and edible MCs which remain attached to the cell surface after differentiation and proliferation [109]. For all of these strategies cells need to adhere to the surface of MCs, for which bench-scale strategies can be similarly applied. The surface of MCs can be modified with proteins such as collagen or fibronectin or conjugated with peptides containing cell adhesion motifs such as the arginine-glycine-aspartate (RGD) sequence, which mediates cell adhesion. These surface modification strategies are currently too expensive for large-scale systems, and the investigation of new, preferably plant sources is warranted. On top of this MCs have the potential to enhance differentiation through the encapsulation and release of appropriate growth or differentiation factors into the cultured tissue.

Several important factors to keep in mind when using microcarriers or other types of supports would be the strength of cell adhesion to their surface, their surface area, porosity, and degradability, and the method for the recovery of microcarriers on a large-scale basis, all of which would influence the design of the bioreactor. An important consideration in the use of MCs for differentiation in a large-scale reactor would be the ability of the MC shape and dimensions to support muscle cell differentiation. Despite culturing in a 3D environment and the curved surface of the MCs, cells have been shown to differentiate on a 2D basis [110]. The proliferation of cells using MCs in suspension cultures is usually associated with the detachment of the cells followed by the seeding of fresh MCs. In general, however, the differentiated phenotype of cells cannot be maintained upon their detachment from the adherent surface [111]. Maintaining the cell phenotype would thus require the use of edible cell culture supports which could then be consolidated, with their adhered content of cells, into the final ‘hybrid’ meat product. Porous microcarriers, although allowing for the good proliferation of cells at first, face difficult issues such as the mass transfer of nutrients and oxygen at later stages of culturing, which affect cell viability [112]. In a similar vein the delivery of growth factors to differentiate the cells may face mass transfer and availability issues. Cell differentiation would also be difficult to monitor when using such microcarriers, which would usually appear too opaque for imaging cells in the interior [113]. An interesting experiment would be the use of high-aspect-ratio MCs and whether these could afford the topographical cues to promote muscle cell alignment as well as myotube and hence myofiber formation. Regardless of the approach the biodegradability and recovery of microcarriers are important aspects in large-scale systems where life cycle assessments should be conducted. In the ideal scenario the microcarriers would be edible, degradable, and/or recycled easily.

### 7.2. Other Cell Culture Supports

Scaffolds and other support configurations possess larger dimensions which may render them more amenable to modifications that promote muscle cell differentiation compared to microcarriers; this is especially true for the case of mechanical stimuli. For example, one company (Mosa Meats) has employed a technology to grow cultured meat by mechanical stimulation based on technology invented by Post and co-workers [114]. In essence, the technology includes the use of pillars with circumferential throughs which scaffold the formation of a biomaterial hydrogel (such as collagen) containing muscle cells. Upon culturing in a suitable differentiation medium, the muscle cells self-assemble to form rings which contract and apply mechanical stresses upon themselves, thus promoting skeletal muscle maturation. As for bench-scale experiments where fluid shear stresses are generally easier to apply than dynamic mechanical stimuli [115], strategies which involve the mechanical motion of the supports themselves would be presumably more difficult to implement for large-scale systems. An easier alternative may be the application of mechanical stresses on cells by employing bioreactor configurations that generate fluid flow patterns in conjunction with appropriate scaffold designs.

As mentioned earlier in this review scaffolds may help in muscle formation by promoting muscle cell adhesion, proliferation, alignment, and differentiation into structured tissue. Looking from a large-scale perspective, the design of the scaffold should be able to accommodate the different stages of muscle cell development. A scaffold used for cell proliferation would require an adequate surface area, either external or internal (for porous substrates), to accommodate the expected increase in the number and volume of muscle cells. On the other hand, scaffolds meant for muscle differentiation should possess the appropriate structure and be imbued with the correct physical and biochemical cues to induce differentiation. For example, the effect of matrix stiffness on differentiation can be practiced either in the form of coatings or the material of the cell support itself. These considerations would in turn have a large effect on the ultimate design of the bioreactor.

### 7.3. Other Factors in the Scaling-Up of Muscle Differentiation Systems

#### 7.3.1. Genetic Modifications of Cells for Large-Scale Cell Proliferation and Differentiation

Cells have a limited capacity to divide, known as the Hayflick limit [116]. Their proliferative capacity can be overcome through genetic modification: for example, cells may be transfected with telomerase, thereby increasing the number of divisions that they are capable of before becoming senescent [117]. This would be important for the large-scale cultivation of cells in particular, where a large passage number would be required. For cellular differentiation transcription factors may be added to cell lines, allowing the cells to follow alternative cell differentiation and renewal processes. As these transcription factors may be incorporated into the genome, resulting in genomic instability, the intermediate and final products arising from the proliferation and differentiation of any type of genetically modified cells must therefore be carefully evaluated to address the safety aspect.

#### 7.3.2. Differentiation Mediums for Large-Scale Reactions

For the proliferation and differentiation of muscle cells the use of two separate mediums is needed. These must be provided to cells in a sequential manner, which may pose difficulties in a biomanufacturing process as any media change would be associated with agitation of the liquid and additional stresses to the cells. The two processes of proliferation and differentiation must be tightly monitored as any imbalance would lead to failure in obtaining the required end product and, consequently, resource wastage. Additionally, suitable mediums for muscle differentiation must be prepared on a large scale. Animal-derived serums, such as horse serum, have been employed for the differentiation of muscle cells into myotubes and eventually into muscle tissue [118]. However, animal-derived sera are deemed to be cruelly obtained and more humane alternatives are soughtOne recent development in the area is serum-free media, which may very possibly replace animal-derived serum in the future. Serum-free media are essentially composed of vitamins, nucleic acid bases, and inorganic compounds that are provided for by a basal medium, e.g., Dubellco’s Modified Eagles Medium (DMEM), mitogens and growth factors, differentiation factors, and components to replace the albumin and protein nutrients in serum [119]. Insulin is a common mitogen that is present in serum and often substituted for in serum-free formulations. This insulin was initially animal-derived [120] but was supplanted by recombinant insulin when it became available [121]. Alternatives to insulin such as insulin mimetics [122] and IGF-1 [123,124] have been investigated. In fact, IGF-1 and not insulin is the native ligand of the IGF receptor and is an effective mitogen at much lower (physiological) concentrations than the latter. In addition to promoting cell proliferation IGF-1 plays a role in regulating skeletal muscle differentiation, where its action is mediated via the IGF-1-Akt signaling pathway [125]. Some of the differentiation factors can be produced recombinantly or replaced by small molecules that activate the same signaling pathways, leading to differentiation. For example, small molecules have been identified which induce Pax3 mRNA during embryoid body differentiation, leading to subsequent skeletal muscle differentiation [126]. Alternatively, activation of the Wnt/beta-catenin pathway can also induce the various events of muscle differentiation, such as cell proliferation, myoblast fusion, and homeostasis [127]. Thus, instead of using Wnt proteins, small molecules that activate the Wnt/beta-catenin pathway such as lithium chloride, CHIR99021, SB-216763, and BIO could potentially be used to promote skeletal muscle formation [128]. The albumin present in the serum fraction of conventional tissue culture media serves to protect cells grown in suspension from shear damage and transport substances such as hormones and drugs to cells in cultures [129]. In serum-free cultures PEG and pluronics can protect cells from shear stresses via mechanisms whereby the polymer forms a protective layer on the cell membrane [130], while the transport function of albumin may be substituted with other proteins from plant sources such as albumin storage proteins from the seeds of dicotyledonous plants [131]. For protein nutrients hydrolysates can be obtained by treating plant protein isolates with microbial enzyme preparations that break down the protein. For example, a chickpea protein hydrolysate could substitute for serum in supporting the growth of monocytic THP-1 cells [132].

The mass transfer of oxygen, nutrients, growth factors, and other proliferation/differentiation factors poses one of the major challenges in bioreactors. While the sparging of oxygen is important for the mass aeration of cells in bioreactors the mammalian cells that are relevant to cultured meat lack cell walls, adhere to the air bubbles, and experience hydromechanical stress, which compromise their viability. Sparge stones and enriched oxygen are thought to curb this issue, with experiments showing an increase in mass transfer and less foaming, which also have an impact on the other factors that are required by the cells. Typically, bioreactors are either stirred tanks or wave bioreactors which make use of rocking motions to facilitate the growth of cultured meat [133]. The design of stirred tanks and wave bioreactors can further help to alleviate the complications of mass transfer.

## 8. Moving towards Cultivated Meat

Given the numerous benefits of lab-grown meat it is no wonder that multiple companies and startups have ventured into the production of cultivated meat after acquiring the appropriate technologies. In terms of technology the commercial interest in cultivated meat is spurring research efforts on several fronts. These include the sourcing and culturing of stem or progenitor cells from suitable species, the development of culture media, the scaling-up of cell expansion and differentiation processes, and the development of scaffolds, the latter which has been discussed in considerable depth in the current review. Being amongst the most well-funded, Upside Foods and Mosa Meat are examples of startups that are building a large cultured meat technology portfolio. On top of producing cultured meat out of myogenic satellite cells, these companies are developing fetal bovine serum (FBS)-free growth media with the hope of greater consumer acceptance [134,135]. Just Eat became the first cultivated meat startup to gain regulatory approval and sell its cultured chicken meat for consumption, in Singapore [136]. Early products, however, are most likely to be in a comminuted form, put together into patties or whole-meat forms (strips, slices) using food-grade binders. On the other hand, the Israeli startup AlephFarms has placed its bet on a technology for creating whole-meat cuts or slices based on a textured soy protein (TSP) scaffold. In one of the handful of publications related to technologies that are being commercialized Ben Arye et al. reported the use of TSP as a scaffold for the seeding of satellite cells, smooth muscle cells, and endothelial cells, all of bovine origin [137]. TSP, produced by a process of low-moisture extrusion, afforded a porous structure that supported good proliferation and differentiation of the cells. To provide a 3D matrix amenable to muscle differentiation and myotube formation fibrinogen was infused into the 3D structure and incubated at 37 °C for gelation of the fibrin. After a three-week period of cell culturing ECM deposition and skeletal muscle maturation were observed, as revealed by proteome analysis.

Not to be outdone, many existing multinational food corporations have also joined the efforts to produce cultured meat. To name but a few, Nestle is partnering with Future Meat Technologies to create a protein blend made up of a mixture of both plant-based and cultured meat [138], while Givaudan, Bühler, and Migros have jointly set up a Cultured Food Innovation Hub for the research and innovation of cultured meat products [139]. Given such enthusiasm from various reputable companies and startups, cultured meat may likely enter the market on a massive scale in due time.

## 9. Conclusions

In the final analysis many considerations are warranted in translating muscle cell differentiation from the micro- to the macroscale. For the most part the incompatibility between the two stems from the fact that microscale systems have relied largely on conventional multiwell tissue culture plates, and as such the generated muscle tissues are not free-standing structures. Therefore, scaffold structures play an important role in bridging microscale and macroscale systems. As an analogy to the native muscle connective tissue scaffolds define the size and shape for muscle tissue formation while presenting the mechanical and biochemical factors essential for muscle cell differentiation and maturation. The challenge of scaling-up would thus be reduced to one of multiplying these individual scaffold microenvironments for a larger volume of tissue culture, such as that in suspension bioreactor systems. In doing so, however, it would be imperative to note and address the specific challenges associated with large-scale cell cultures: for example, the mass transfer of growth factors and nutrients as well as the ability of the cell culture supports to withstand the larger shear stresses in the bioreactor. In short, the behavior of the scaffold materials in response to the bioreactor conditions and stimuli has to be re-evaluated, regardless of the conditions optimized for the microsystem.

No doubt in its infancy stages, the cultivation of meat on a large scale is not too far-fetched an idea. From the aspect of muscle cell differentiation many of the relevant technologies have already been developed, especially at the microscale. It is envisioned that the adaptation of these technologies to the challenges at hand will eventually help to realize the viability and value of cultured meat.
